# *α*-Mangostin Improves Glucose Uptake and Inhibits Adipocytes Differentiation in 3T3-L1 Cells via PPAR*γ*, GLUT4, and Leptin Expressions

**DOI:** 10.1155/2015/740238

**Published:** 2015-03-22

**Authors:** Muhammad Taher, Mohamed Zaffar Ali Mohamed Amiroudine, Tengku Muhamad Faris Syafiq Tengku Zakaria, Deny Susanti, Solachuddin J. A. Ichwan, Mohd Arifin Kaderi, Qamar Uddin Ahmed, Zainul Amiruddin Zakaria

**Affiliations:** ^1^Department of Pharmaceutical Technology, Faculty of Pharmacy, International Islamic University Malaysia, Jalan Istana, Bandar Indera Mahkota, 25200 Kuantan, Pahang, Malaysia; ^2^Department of Chemistry, Faculty of Science, International Islamic University Malaysia, Jalan Istana, Bandar Indera Mahkota, 25200 Kuantan, Pahang, Malaysia; ^3^Faculty of Dentistry, International Islamic University Malaysia, Jalan Istana, Bandar Indera Mahkota, 25200 Pahang, Malaysia; ^4^Faculty of Allied Health Science, International Islamic University Malaysia, Jalan Istana, Bandar Indera Mahkota, 25200 Kuantan, Pahang, Malaysia; ^5^Department of Pharmaceutical Chemistry, Faculty of Pharmacy, International Islamic University Malaysia, Jalan Istana, Bandar Indera Mahkota, 25200 Kuantan, Pahang, Malaysia; ^6^Department of Biomedical Sciences, Faculty of Medicine and Health Sciences, Universiti Putra Malaysia, 43400 Serdang, Selangor, Malaysia

## Abstract

Obesity has been often associated with the occurrence of cardiovascular diseases, type 2 diabetes, and cancer. The development of obesity is also accompanied by significant differentiation of preadipocytes into adipocytes. In this study, we investigated the activity of *α*-mangostin, a major xanthone component isolated from the stem bark of *G. malaccensis*, on glucose uptake and adipocyte differentiation of 3T3-L1 cells focusing on PPAR*γ*, GLUT4, and leptin expressions. *α*-Mangostin was found to inhibit cytoplasmic lipid accumulation and adipogenic differentiation. Cells treated with 50 *μ*M of *α*-mangostin reduced intracellular fat accumulation dose-dependently up to 44.4% relative to MDI-treated cells. Analyses of 2-deoxy-D-[^3^H] glucose uptake activity showed that *α*-mangostin significantly improved the glucose uptake (*P* < 0.05) with highest activity found at 25 *μ*M. In addition, *α*-mangostin increased the amount of free fatty acids (FFA) released. The highest glycerol release level was observed at 50 *μ*M of *α*-mangostin. qRT-PCR analysis showed reduced lipid accumulation via inhibition of *PPARγ* gene expression. Induction of glucose uptake and free fatty acid release by *α*-mangostin were accompanied by increasing mRNA expression of *GLUT4* and *leptin*. These evidences propose that *α*-mangostin might be possible candidate for the effective management of obesity in future.

## 1. Introduction

The incidence of obesity has become a major public health concern worldwide. Obesity is characterised by the accumulation and deposition of excess fat in adipose tissues [1] and increased deposition of cytoplasmic triglycerides [2], which may lead to various metabolic and chronic diseases such as cardiovascular diseases, type 2 diabetes, and cancer [3]. The development of obesity in adults is also accompanied by significant differentiation of preadipocytes into adipocytes [4].

In recent years, research studies on adipocytes have been growing tremendously. Accordingly, adipocytes are emerging as a major drug target for diabetes and obesity-mediated metabolic syndrome [5]. Adipose is not only known for its capacity to store the excess of dietary energy in the form of triglyceride [6], but has also been acknowledged to play an important role in the control of energy metabolism [7, 8]. A number of transcription factors have been documented to be involved in the adipogenesis, glucose uptake, and glycolysis pathway [9]. These transcription factors include peroxisome proliferator-activated receptor-*γ* (PPAR*γ*), glucose transporter-4 (GLUT4), and adipokines such as leptin.

PPAR*γ* is predominantly expressed in adipose tissues and plays a central role in adipose tissue functions [10]. PPAR*γ* regulates the expression of genes associated with insulin signalling and glucose and lipid metabolism in mature adipocytes [11]. Reduced expression of PPAR*γ* has been shown to be effective in inhibiting the adipogenesis of 3T3-L1 cells [12]. GLUT4 is a high-affinity glucose transporter predominantly expressed in insulin-sensitive tissues such as muscle and adipocytes [13]. Increased expression and plasma membrane translocation of GLUT4 have been found to lower blood glucose and enhance glucose transport and utilisation [14]. Leptin is produced mainly by adipocytes and found in low levels in the gastric fundic epithelium, intestine, skeletal muscle, mammary epithelium, placenta, and brain [15]. High levels of circulating leptin in adipose tissues characterise human obesity [16] and increased levels of body fat [17]. As a result, the role of some gene expressions and the importance of these expressions have been studied.

A detailed review of the literature shows that a few species of* Garcinia* have attracted the attention of health practitioners [18]. For example, the fruit of* G. cambogia* has been shown to have antiobesity effect [19, 20], affect the lipid metabolism [21], and inhibit the cytoplasmic lipid accumulation as well as adipogenic differentiation of preadipocytes [22]. Moreover, water-soluble calcium hydroxycitrate (HCA) as* G. atroviridis* has been used for the treatment of obese women [23], while* G. indica *(hydroxycitric acid) has been used as a weight loss supplement for obese patients [24]. Due to the presence of active compounds in different* Garcinia* species,* Garcinia* has been regarded as an interesting choice to be studied further. The species of* Garcinia* have been reported to possess a lot of bioactive molecules such as xanthones, flavonoids, benzophenones, lactones, and phenolic acids [25]. Recently, the stem bark of* G. malaccensis* has been shown to contain xanthones, namely, *α*-mangostin as the major compound [26]. Interestingly, the most studied xanthone of* Garcinia* species is *α*-mangostin for which antibacterial [27], antioxidant [28], anti-inflammatory [29–31], antiproliferative [32], proapoptotic [33–35], and anticarcinogenic [36–39] activities have already been reported.

This paper reports the effect of *α*-mangostin isolated from the stem bark of* G. malaccensis *on 3T3-L1 adipocytes via the adipogenesis and glucose uptake activity. In this study, we evaluated the effect of *α*-mangostin on the cell viability based on the MTT assay and quantification of lipid accumulation in 3T3-L1 cells. We also examined insulin-induced glucose uptake into 3T3-L1 adipocytes as a measure by the liquid scintillation counter. Finally, qRT-PCR analysis was performed in order to find out the gene expression responsible for the *α*-mangostin ability to reduce lipid accumulation in adipose tissues. As a result, we demonstrated that *α*-mangostin may reduce lipid accumulation as well as stimulate glucose uptake by the cells and may be a promising candidate as an antiobesity agent.

## 2. Materials and Methods

### 2.1. Plant Materials and Isolation of *α*-Mangostin

**Figure 1:**
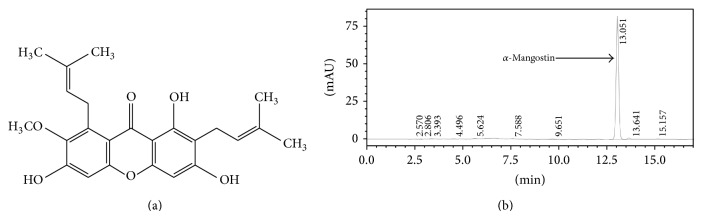
The purity of *α*-mangostin was determined using HPLC analysis. (a) Chemical structure of *α*-mangostin. (b) The amount was measured by HPLC technique. The peak of *α*-mangostin was observed at retention time of 13.05 min.

*α*-Mangostin was isolated from* G. malaccensis* [26]. The purity of the isolated *α*-mangostin was approximately 94% as identified by HPLC (Figure 1) and compared to the reference standard of purity 96.5% of *α*-mangostin (C_24_H_26_O_6_) from ChromaDex (Irvin, CA) [40]. Mouse 3T3-L1 fibroblast (CL-173) was obtained from the American Type Culture Collection (ATCC), Virginia, USA.

### 2.2. HPLC Analysis

Quantification of *α*-mangostin was performed according to method described by Elsaid Ali et al. [40]. The HPLC system (Shimadzu, Japan) consisted of a quaternary pump (LC-20AT), autosampler (SIL-20A HT), solvent degasser unit (DGU-20A 5R), diode array detector (SPD-M20A), and column oven (CTO-10AS VP). The quantification wavelength of *α*-mangostin was set at 316 nm. The chromatographic separation was performed at ambient temperature (25–28°C) using Hypersil BDS C_18_ column (4.6 × 100 mm, 3 *μ*m size) (Thermo Scientific, USA) with C_18_ guard column. The mobile phase consisted of acetonitrile (A) and 0.1% (v/v) orthophosphoric acid (H_3_PO_4_) diluted in water (B) was delivered at flow rate of 1.0 mL/min following programmed gradient elution: 70% (A) isocratic for 6 min, 70–75% (A) in 1.2 min, 75–80% (A) in 0.4 min, 80% (A) isocratic for 2.4 min, 80–70% (A) in 0.4 min, and finally 70% (A) isocratic for 5 min as postrun for reconditioning. Sample injection volume was adjusted to 10 *μ*L. Total running time was 17 min. All solutions of mobile phase were freshly prepared, filtered through 0.45 *μ*m Nylon filter under vacuum, and degassed by sonication for 20 min prior to use. The results were analysed using ChemStation software. The percentage of *α*-mangostin in the extract was calculated based on the peak area.

### 2.3. Cell Culture

3T3-L1 preadipocytes were grown in Dulbecco's Modified Eagle Media (DMEM) containing 10% foetal bovine serum (FBS), 1% penicillin (10.000 U/mL), and 1% streptomycin (10.000 *μ*g/mL supplemented in 37°C incubator in a humidified atmosphere of 5% CO_2_). Cells were subcultured every 3 to 4 days at approximately 80% confluence.

### 2.4. Cell Viability

Cell viability was assessed by 3-(4,5-dimethylthiazol-2-yl)-2,5-diphenyltetrazolium bromide (MTT) assay. Mature adipocytes were seeded in 96-well plates and grown until confluence. *α*-Mangostin was dissolved in dimethyl sulfoxide (DMSO) and treated for 48 h. Our preliminary study showed that DMSO at a final concentration of <0.1% in media did not affect cell viability or differentiation. Cells were then washed two times with phosphate buffer saline (PBS). 20 *μ*L of MTT stock solution (5 mg/mL) was added to each well and the plates were further incubated for 4 h at 37°C. 100 *μ*L of DMSO was added to each well to solubilise the water-insoluble purple formazan crystals [41]. After 1 h, the absorbency was measured at wavelength of 570 nm and reference wavelength of 630 nm with a microplate reader.

### 2.5. Adipocyte Differentiation

Cells were seeded onto 12-well plates at a density of 2 × 10^4^ cells/well. Two days after confluence (defined as day-0), cells were stimulated to differentiate with differentiation medium containing DMEM with 10% FBS and MDI [0.5 mM 3-isobutyl-1-methylxanthine (IBMX), 0.25 *μ*M dexamethasone, and 1 *μ*g/mL insulin] for 2 days. In the course of screening adipocyte differentiation-inhibitory activity, 3T3-L1 preadipocytes were treated with differentiation medium in the presence of various concentrations of test compound (10, 25, and 50 *μ*M of *α*-mangostin) at day-0. At day-2, differentiating medium was replaced with 10% FBS/DMEM medium containing 1 *μ*g/mL insulin and incubated for another two days (day-4). Thereafter, the cells were maintained in 10% FBS/DMEM medium for an additional 4 days (day-8) with medium changes every 2 days [42]. Adipocytes differentiation scheme was summarized in Table 1.

### 2.6. Oil Red O Staining

Eight days after the differentiation induction, cells were washed three times with PBS and fixed with 10% formalin for 1 h at room temperature. After fixation, cells were washed once with PBS and stained with freshly diluted Oil Red O solution (3 parts of 0.6% Oil Red O in isopropanol and 2 parts of water) for 1 h. Cells were then washed twice with distilled water and visualised under a microscope. Images were collected on an Olympus (Tokyo, Japan) microscope. For quantitative analysis, Oil Red O stain was dissolved with isopropanol and optical density was measured at 520 nm by enzyme-linked immunosorbent assay (ELISA) plate reader [43].

### 2.7. Deoxy-[^3^H]-D-glucose Uptake Assay

Glucose uptake activity was analysed by measuring the uptake of radiolabelled glucose from the culture medium by adipocytes [42]. Briefly, the differentiated adipocytes, which were grown in 12-well plates, were washed twice with serum-free DMEM and incubated for 3 h at 37°C with 1 mL of serum-free DMEM. The cells were then washed three times with Krebs-Ringer HEPES (KRPH) buffer (118 mM NaCl, 5 mM KCl, 1.3 mM CaCl_2_, 1.2 mM MgSO_4_, 1.2 mM KH_2_PO_4_, and 30 mM HEPES, pH 7.4) and incubated with 0.9 mL of KRPH buffer for 30 min at 37°C. *α*-Mangostin (10, 25, and 50 *μ*M) including the control was added, and the cells were incubated at 37°C for further 60 min. Glucose uptake was initiated by the addition of 0.1 mL of KRPH buffer containing 2-deoxy-D-[^3^H]-glucose (0.037 MBq; Perkin Elmer Inc., USA) and glucose (0.001 mM). After 60 min, the cells were washed three times with ice-cold PBSA to stop the glucose uptake. The cells were then lysed through incubation for 20 min at 37°C with 0.7 mL of 1% Triton X-100. The radioactivity levels in the cell lysates were determined using a Tri-Carb 2700TR liquid scintillation counter, Packard Instrument Co. (USA). Samples from each lysate were counted and measured in triplicate.

### 2.8. Adipolysis Assay

Adipolysis assay kits (Cayman Chemical, USA) was used to study the adipolysis of triglycerides in differentiated 3T3-L1 cells. In order to perform the assay, the culture medium was carefully aspirated and replaced with fresh medium containing the various concentrations of *α*-mangostin (10, 20, and 50 *μ*M). The isoproterenol solution was used as a positive control. Test compound (*α*-mangostin) and positive control were used to induce adipolysis and the amount of detectable free glycerol depend on the activity of the samples. Absorbance was finally measured at 540 nm.

### 2.9. Quantitative Reverse Transcriptase Polymerase Chain Reaction (qRT-PCR) Analysis

Total RNA was extracted using Trizol reagent (Invitrogen, USA). The mRNA in the samples was reverse-transcribed into cDNA using QuantiTect Reverse Transcription (Qiagen) kit according to the manufacturer's instructions. The resulting cDNA was amplified by PCR using the following primer pairs: mouse* PPARγ* (sense 5-TTTTCAAGGGTGCCAGTTTC-3 and antisense 5-AATCCTTGGCCCTCTGAGAT-3),* GLUT4* (sense 5-CAGCTCTCAGGCATCAAT-3 and antisense 5-TCTACTAAGAGCACCGAG-3), and* leptin* (sense 5-GGAGGAATCCCTGCTCCAGC-3 and antisense 5-CTTCTCCTGAGGATACCTGG-3). The housekeeping beta-actin gene was amplified using the sense primer 5-ACACCCCAGCCATGTACG-3 and the antisense primer 5-TGGTGGTGAAGCTGTAGCC-3.

### 2.10. Statistical Analysis

Data are presented as means ± standard error of three experiments. Data were analysed by ANOVA using SPPS version 19. A *P*-value of less than 0.05 was considered statistically significant.

## 3. Results and Discussion

### 3.1. Effect of *α*-Mangostin on the Viability of 3T3-L1 Cells

The viability assay was used to determine any possible adverse effects of *α*-mangostin (isolated from* G. malaccensis*) on the cells. The concentrations of 0, 10, 20, and 50 *μ*M had no effect on the cell viability. Based on the MTT assay, the dosage of 100 *μ*M significantly (*P* < 0.05) decreased the cell viability and therefore was not used as treatment dose in this study.

### 3.2. Reduction of Intracellular Lipid Accumulation in 3T3-L1 Cells by *α*-Mangostin

**Figure 2:**
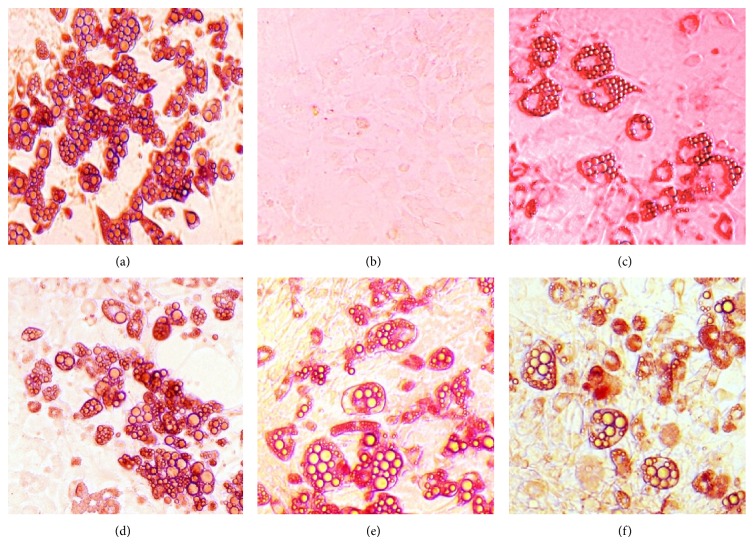
Stained fat droplets after differentiation programme (magnification 200x). (a) Insulin. (b) DMSO (negative control). (c) MDI-treated cells. ((d)–(f)) *α*-Mangostin, 10, 25, and 50 *μ*M, respectively.

**Figure 3:**
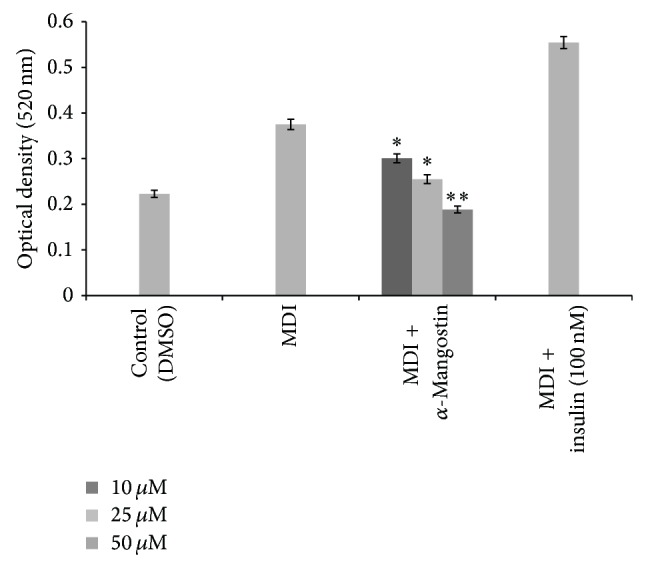
Effect of different concentration of *α*-mangostin (10, 25, 50 *μ*M) on the differentiation of 3T3-L1 adipocytes. Data is represented as mean ± SD, with *n* = 3 per group. ^*^ *P* < 0.05, ^**^ *P* < 0.01 compared to MDI-treated cells.

Eight days after treatment, preadipocytes differentiation was terminated and stained with Oil Red O. Fat droplets in these cells were visualised and photographed (Figure 2). Quantification of lipid accumulation by using UV spectrophotometer at 520 nm showed that cells treated with *α*-mangostin reduced intracellular fat accumulation dose-dependently up to 44.4% relative to MDI-treated control cells at the dose of 50 *μ*M (Figure 3). Therefore, our results demonstrated that *α*-mangostin added to the adipocyte inducer (IBMX, dexamethasone, and insulin) showed antiadipogenic activity as evidenced by decreased triglyceride accumulation in 3T3-L1 cells at all of the tested concentrations. This unique mechanism of decreased lipid formation [19] by *α*-mangostin further prompted us to study the characteristic effect of *α*-mangostin on adipocyte differentiation by using the quantitative RT-PCR. Since most studies have shown that* PPARγ* is one of the target genes in the induction of adipocyte differentiation [11], owing to the same fact, the same gene was used to determine the mechanism for the inhibitory effect of *α*-mangostin on the 3T3-L1 cells.

### 3.3. Stimulation of Glucose Uptake in Mature 3T3-L1 Adipocytes by *α*-Mangostin

**Figure 4:**
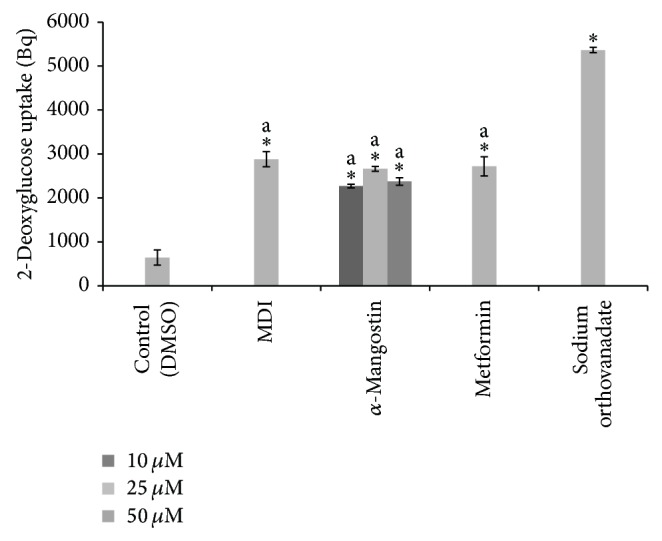
The effects of different concentration of *α*-mangostin (10, 25, 50 *μ*M) in glucose uptake of 3T3-L1 adipocytes. Data is represented as mean ± SD, with *n* = 3 per group. ^*^ *P* < 0.05 compared to control group (DMSO treated cells), a = significant at indicated concentration.

As PPAR*γ* ligands can affect the adipocyte differentiation and are reported to have an effect on glucose uptake in 3T3-L1 adipocytes [44], hence, the next part of this study was designed to evaluate the effect of *α*-mangostin on the glucose uptake and insulin sensitivity. To determine 2-deoxy-D-glucose (2-DG) uptake stimulation by 3T3-L1 adipocytes, mature 3T3-L1 adipocytes were treated with *α*-mangostin at the indicated concentrations (10, 25, and 50 *μ*M) for 60 min, and then glucose uptake activity was assessed. The cells were treated with metformin (1 mM) and sodium orthovanadate (5 mM) as positive controls, and undifferentiated DMSO treated cells were used as a negative control for the assay. The results indicated that the *α*-mangostin stimulated the glucose uptake in 3T3-L1 adipocytes. However, the insulin-induced glucose uptake was decreased by *α*-mangostin at 50 *μ*M. This finding was interesting since the test compound also reduced lipid formation at the same dose for up to 44.4% compared to the MDI-treated cells. This raised our attention to further evaluate the mechanism of related mRNA expression.  Figure 4 illustrates the insulin-stimulated glucose uptake by the compounds. Levels of radioactivity in the cell lysates were determined using a liquid scintillation counter.

Adipocyte formation (adipogenesis), which occurs in several stages, is the development of mature fat cells from preadipocytes. This process includes alteration of cell shape, growth arrest, clonal expansion, and a complex sequence of changes in gene expression and storage of lipid [45]. Based on the above findings, it appears that our findings related to the effect of *α*-mangostin on 3T3-L1 adipocytes were in agreement with several other reports [46, 47] stating that compound that inhibits adipocyte differentiation can also improve glucose uptake in the 3T3-L1 adipocytes.

In adipocytes, basal (cells treated with normal glucose without the presence of insulin and 2-deoxy-D-[^3^H]-glucose) and insulin-stimulated glucose uptake activity require a glucose transporter. Insulin can accelerate glucose entry by affecting the translocation of GLUT4 from intracellular stores to the plasma membrane [48]. In general, it is known that GLUT4 provides insulin-stimulated glucose transport in adipocytes [49]. Moreover, GLUT4 is expressed only in adipocytes and its expression is regulated by PPAR*γ* [50]. Therefore, to go for any conclusion in this study, it seemed crucial to evaluate the expression of these genes in order to understand the mechanism of glucose uptake in the 3T3-L1 adipocytes.

### 3.4. Induction of Adipolysis by *α*-Mangostin

**Figure 5:**
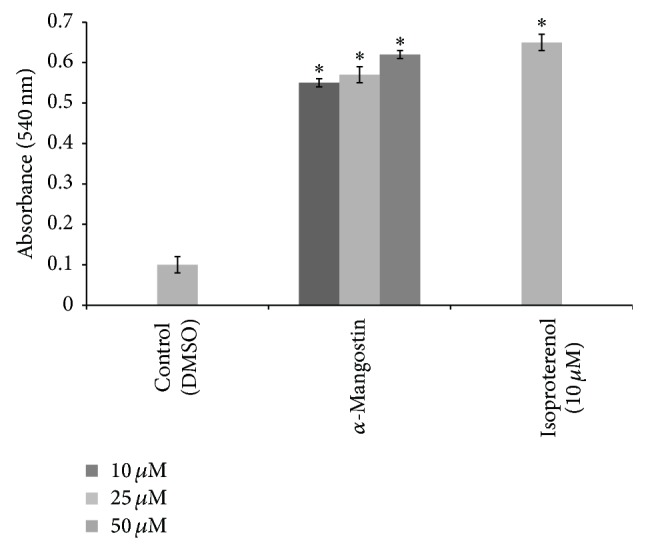
Glycerol release of different concentration of *α*-mangostin (10, 25, 50 *μ*M) from the 3T3-L1 cells. Data is represented as mean ± SD, with *n* = 3 per group. ^*^ *P* < 0.05 compared to control group (DMSO treated cells).

Figure 5 shows that the adipolysis assay served as confirmatory results for the adipogenesis assay. The amount of glycerol released into the medium was proportional to the level of triglyceride storage and degree of adipolysis. It was found that cells treated with *α*-mangostin increased the amount of free fatty acid released into the medium. It was further observed that the *α*-mangostin result was similar with the isoproterenol (10 *μ*M), which was used as the positive control for the study. Lipolysis was measured by quantifying the glycerol released into the medium. Lipolysis in adipocytes is modulated in a stepwise fashion by several genes including the* leptin*. After this gene initiates the lipolysis pathway by releasing the fatty acids, glycerols are also released [51]. Moreover, it has been reported that plant extracts that inhibit the adipocyte differentiation of 3T3-L1 cells will also decrease the serum triglyceride levels in the adipose tissue [52]. These evidences show that decreased adipocytes differentiation may trigger the adipocytes to release the triglyceride into the medium.

Generally, triglyceride can be converted into glycerol and free fatty acid when released into the medium. The data from the study showed that *α*-mangostin enhanced lipolysis, and the efficacy of this action improved proportionally with the increase in concentration of *α*-mangostin. The highest glycerol release level was observed for cells treated with *α*-mangostin at 50 *μ*M.

### 3.5. Inhibition of 3T3-L1 Adipocyte Differentiation Involving PPAR*γ*

**Figure 6:**
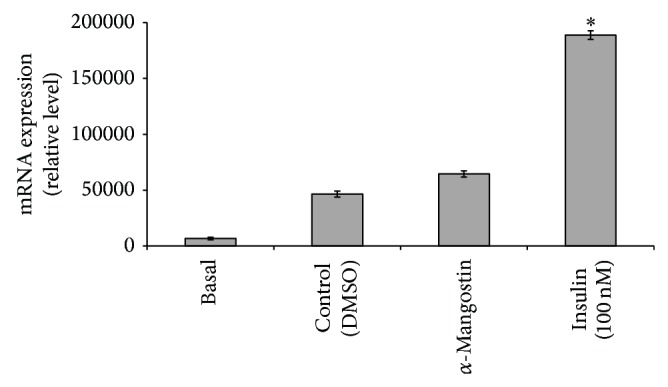
Effects of *α*-mangostin (25 *μ*M) on* PPARy* mRNA expression. Data is represented as mean ± SD, with *n* = 3 per group. ^*^ *P* < 0.05 compared to control group (DMSO treated cells).

To further assess the activity of *α*-mangostin on* PPARγ * mRNA expression, 3T3-L1 preadipocytes were cultured with the indicated concentration of the compound (25 *μ*M) in the presence of MDI. This method can evaluate the stimulation or inhibitory effect of the test compound on adipocyte differentiation. As shown in Figure 6, cells treated with *α*-mangostin showed reduced activity of* PPARγ* level when compared with the basal. Insulin (100 nM) was used as the positive control [53]. Increased expression of PPAR* γ* induces adipocyte differentiation in 3T3-L1 cells whereas the suppression of PPAR *γ* expression blocks the lipid formation [12]. In this study, analysis of the gene expression demonstrated that the 3T3-L1 cells treated with *α*-mangostin significantly downregulated the* PPARγ* expression. Therefore, we can deduce that the reduction in* PPARγ* may be the key step in the inhibition of adipocyte differentiation [22] in 3T3-L1 and that the targeting of PPAR *γ* could be the main mechanism for blocking of adipocyte differentiation by the *α*-mangostin.

### 3.6. Upregulation of Transcription of GLUT4 in 3T3-L1 Cells by *α*-Mangostin

**Figure 7:**
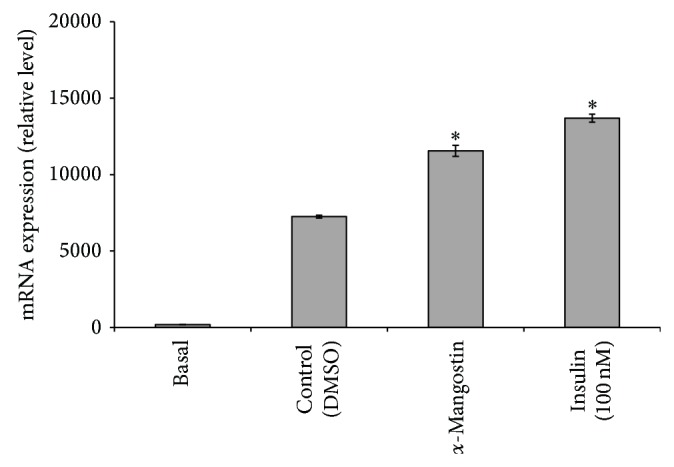
Effects of *α*-mangostin (25 *μ*M) on* GLUT4* mRNA expression. Data is represented as mean ± SD, with *n* = 3 per group. ^*^ *P* < 0.05 compared to control group (DMSO treated cells).

Afterward, we investigated the effects of *α*-mangostin on the* GLUT4 *mRNA expression. As mentioned previously, the glucose uptake activity in adipocytes is related to the expression of glucose transporters. In this study, it was observed that all the* GLUT4* mRNA expression in fully differentiated adipocytes increased significantly after treatment with *α*-mangostin (Figure 7). This result demonstrated that the *α*-mangostin promoted glucose uptake in 3T3-L1 adipocytes via the upregulation of GLUT4 expression.

GLUT4, which transports glucose from blood into tissue, is the principal glucose transporter among several isotypes of glucose transporters in insulin-sensitive tissues such as skeletal muscle and adipocytes [47]. Decrease in the translocation of GLUT4 to the plasma membrane has been found to be the major cause of insulin resistance [46], and it is required to activate GLUT4 in skeletal muscle to improve insulin resistance and to maintain blood glucose homeostasis. Metformin, which is one of the widely used antidiabetic drugs, can enhance the insulin-stimulated glucose uptake by increasing the cell surface GLUT4 content [11]. In this study, to assess whether the increased glucose uptake stimulated by *α*-mangostin was due to the translocation of GLUT4, the amount of* GLUT4* expression present in the cells was measured by using the quantitative qRT-PCR.

As shown previously, the *α*-mangostin treatment significantly (*P* < 0.05) upregulated the mRNA expression of* GLUT4 *gene in fully differentiated adipocytes. This result indicates that the *α*-mangostin accelerated basal and insulin-mediated glucose uptake by upregulating the GLUT4 expression. Decreased GLUT4 expression in adipose tissue is associated with obesity and type 2 diabetes in humans [54]. Thus, it is important to use a compound that could improve glucose uptake and stimulate GLUT4 expression.

### 3.7. Role of *α*-Mangostin in Leptin Expression

**Figure 8:**
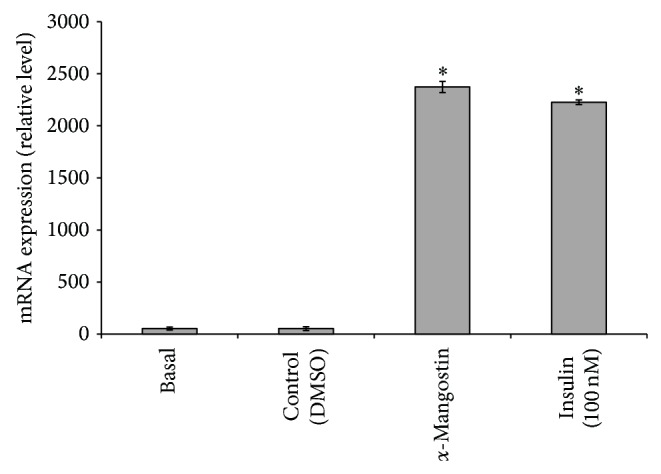
Effects of *α*-mangostin (25 *μ*M) on* leptin* mRNA expression. Data is represented as mean ± SD, with *n* = 3 per group. ^*^ *P* < 0.05 compared to control group (DMSO treated cells).

Finally, in the gene expression analysis, we evaluated the effect of *α*-mangostin on* leptin* expression to signify the free fatty acid release from the cells into the medium. Analysis with *α*-mangostin (25 *μ*M) showed that the compound increased the* leptin* expression and the result was similar with insulin (100 nM) that was used as the positive control for the study (Figure 8). This result suggests that *α*-mangostin treatment may release the free fatty acid into the medium, which was confirmed by the* leptin* mRNA expression. In obese individuals, plasma leptin and free fatty acid are both elevated. Since free fatty acid also reduces plasma leptin levels in the* in vitro* study [5], it has been speculated that obesity may be caused by the abnormality in the leptin-reduction mechanism of free fatty acids. Alternatively, leptin resistance, which has been considered to be present in obesity, might overcome the decrease in leptin induced by free fatty acid [55].

## 4. Conclusion

In summary, the present study has shown that *α*-mangostin isolated from* G. malaccensis* reduces lipid accumulation with decreased* PPARγ* expression as well as stimulating the glucose uptake and free fatty acid release from the cells via* GLUT4* and* leptin* expression. Taken together, these results indicate that *α*-mangostin derived from* G. malaccensis *may be a candidate for preventing metabolic disorders such as obesity particularly among diabetics.

## Figures and Tables

**Table 1:** Differentiation scheme of adipocytes.

Day	Negative control	Positive control	Treatment group
0	No inducer (solvent 0.1% ethanol)	Dexamethasone (0.25 *μ*M) IBMX (0.5 mM) Insulin (1.0 *μ*g/mL)	Dexamethasone (0.25 *μ*M) IBMX (0.5 mM) *α*-Mangostin (10, 20, 50 *μ*M)

2	No inducer (solvent 0.1% ethanol)	Insulin (100 nM)	Insulin (100 nM)

4–8	Complete medium	Complete medium	Complete medium
